# Locating hydrogen in the Mg_5_Bi_3_H_x_ Zintl phase

**DOI:** 10.1038/s42004-025-01530-1

**Published:** 2025-04-30

**Authors:** Teuta Neziraj, Lev Akselrud, Marcus Schmidt, Ulrich Burkhardt, Yuri Grin, Ulrich Schwarz

**Affiliations:** 1 Max-Planck-Institut für Chemische Physik fester Stoffe, Dresden, Germany; 2 Ivan Franko National University of Lviv, Lviv, Ukraine

**Keywords:** Solid-state chemistry, Chemical bonding

## Abstract

The preparation of Zintl phases with pronounced spin-orbit coupling has received substantial scientific interest because of their distinctive electronic properties. In the context of superconductivity and topological phenomena related to band inversion, intermetallic compounds of bismuth have come into focus recently. While bismuth forms a rich variety of Zintl phases with the heavier alkaline-earth metals, there are significantly fewer magnesium compounds. Here we show that high-temperature high-pressure synthesis opens a convenient route for the preparation of Mg_5_Bi_3_H_x_ already at moderate conditions. The compound (space group *Pnma, a* = 11.5399(3) Å, *b* = 8.9503(2) Å and *c* = 7.8770(2) Å) adopts a Ca_5_Sb_3_F crystal structure. The minute amounts of hydrogen could only be detected by thermal decomposition of the compound in combination with mass spectroscopy of the gas phase. Direct space analysis of the chemical bonding allowed for allocating the hydrogen position at a partially occupied interstitial site and reveals strongly polar Mg-Bi and Mg-H bonds in accordance with the Zintl concept. Calculated band structures exhibit substantial electronic reorganization upon hydrogen insertion. The combination of advanced analytical tools in concert with modern quantum chemical techniques provides an efficient approach to allocate trace amounts of interstitial atoms stabilizing intermetallic phases.

## Abstract (web summary)

Intermetallic compounds of bismuth exhibit superconductivity and topological phenomena, with their formation under extreme conditions having been extensively investigated. Here, the authors report the synthesis of Mg_5_Bi_3_H_x_ at 4–5 GPa and 973–1273 K, observing H at partially occupied interstitial positions, following the Zintl concept.

## Introduction

The renewed interest in intermetallic compounds of bismuth is driven by the frequent occurrence of valuable physical properties like superconductivity^1–3^ and the recurrent appearance of topological phenomena, which originate from band inversion resulting from the enormous spin-orbit coupling in the heavy element^4–6^. Intermetallic compounds of bismuth already attracted the attention of Zintl^7,8^ who recognized that the salt-like properties of Mg_3_Bi_2_ may be described by the electron-precise balance [Mg^+2^]_3_[Bi^−3^]_2_. Despite the later discovery of numerous new bismuth phases of other alkaline-earth metals^9^, Mg_3_Bi_2_ remained the only compound in the system Mg-Bi. Its ambient-temperature modification transforms into a disordered high-temperature form at 959 K^10^.

In recent studies, the formation of new bismuth compounds has been extensively investigated at extreme conditions. The discovery of several new binary phases with transition-metals^11–17^ suggests that application of high pressure is a suitable means to manufacture unacquainted products. The present study in the system magnesium—bismuth at high-pressure high-temperature conditions discloses a compound with composition Mg_5_Bi_3_H_x_ containing only approximately 0.03 wt% hydrogen. Diligent investigation of the reaction conditions verify that the formation of the compound requires the presence of those infinitesimal amounts of hydrogen. Consequently, the atomic arrangement is assigned to the Ca_5_Sb_3_F type^18,19^. As a result of the present study, we report the discovery of the ternary phase Mg_5_Bi_3_H_x_. A detailed analysis of the chemical bonding sheds light on the atomic charges as well as on the position of the interstitial hydrogen and its role for the stability of the phase. The band structure changes upon hydrogen insertion involve sophisticated charge adjustments within the polar intermetallic compound.

## Results and discussion

### Synthesis and characterization

The maximum yield of the new compound is achieved by treatment of a mixture Bi_62.5_Mg_37.5_ at 4 GPa combined with heating for 30 min at 1073(50) K followed by annealing for 3 h at 773(30) K before quenching under load. Powder X-ray diffraction patterns of the product show reflections of the new compound plus a second set, which is compatible with the well-known Mg_3_Bi_2_-type crystal structure.

The phases in the product mixture reveal insignificant (Supplementary Fig. 1) X-ray backscattering contrast with an average composition amounting to Mg_62.8(5)_Bi_37.2_ of metallographic samples (Supplementary Fig. 2). We note here that the accompanying side phase, which is commonly labelled as Mg_3_Bi_2_, exhibits a broad homogeneity range from Mg_60_Bi_40_ up to Mg_64_Bi_36_ at ambient pressure and 773 K^20–22^.

**Fig. 1:**
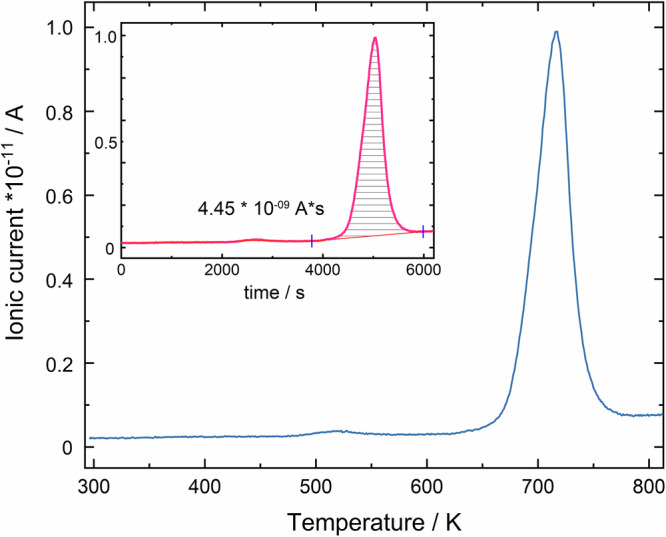
Hydrogen signal H_2_^+^ of the mass spectrometer. (Main panel) Measured ionic current as a function of temperature. (Inset) Signal of the ionic current as a function of time and integral (shaded area) for the determination of the total charge, which is linearly proportional to the amount of hydrogen.

For further chemical analysis, the mass loss of an as-cast mixture from the high-pressure synthesis is investigated by ramping the temperature and performing mass spectroscopy of the gas phase (Fig. 1). The measurements indicate that 23.9 mg of sample contain 0.0046 mg of hydrogen, which is released at temperatures above 613 K (onset). Using the phase amount of Mg_5_Bi_3_H_x_ as determined by X-ray powder refinements (approximately 80 mass%) and the determined charge, the hydrogen content of the product mixture (0.019 mass%) is estimated by a calibration function (Supplementary Fig. 3). The composition of the new phase amounts to Mg_5_Bi_3_H_x_ with *x* ≈ 0.2. Chemical analysis of the elements used for synthesis clearly identified significant amounts of hydrogen in the magnesium and bismuth powders used for synthesis (Supplementary Fig. 4). Using carefully dehydrogenated magnesium and bismuth fail to yield the new ternary phase pointing at an essential role of hydrogen for stabilization of the new compound.

**Fig. 2:**
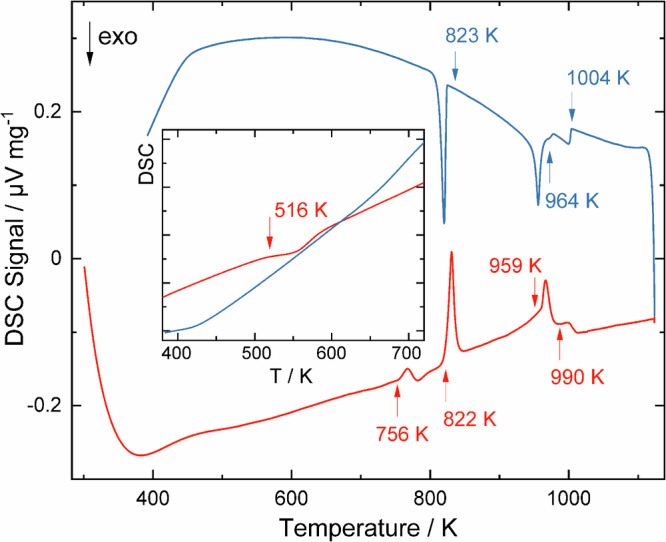
Differential scanning calorimetry measurement of the product from high-pressure synthesis. The phase mixture contains Mg_3_Bi_2_ and the new phase Mg_5_Bi_3_H_x._ The exothermal signal in the inset is attributed to the decomposition of Mg_5_Bi_3_H_x_ into Mg_3_Bi_2_ and Mg(Bi). All effects occurring at higher temperatures are endothermal upon heating in accordance with the phase diagram refs. 20–22. The signal at 756 K is assigned to the melting of Mg(Bi), the one at 822 K is attributed to the melting of the eutectic mixture. At 959 K the transformation of the low-temperature modification of Mg_3_Bi_2_ into the high-temperature form is indicated, and 990 K corresponds to the liquidus.

Upon heating at ambient pressure, the as-cast high-pressure product exhibits an exothermal, monotropic effect at approximately 516(10) K in differential scanning calorimetry measurements (Fig. 2). After the thermal treatment, the reflections of the new phase Mg_5_Bi_3_H_x_ have disappeared and powder X-ray diffraction data essentially indicate a transformation of the hydride into Mg_3_Bi_2_ (Supplementary Fig. 5). These findings indicate that the compound Mg_5_Bi_3_H_x_ is a metastable high-pressure phase.

Structure solution and refinement of the high-pressure product are done using powder X-ray diffraction data. Indexing of the reflections of the new phase suggests orthorhombic symmetry with systematic extinctions being compatible with the space groups *Pnma* and *P*2*na*. The intensity pattern points at a crystal structure which is related to the *β*-Yb_5_Sb_3_-type (Supplementary Fig. 6).

**Fig. 3:**
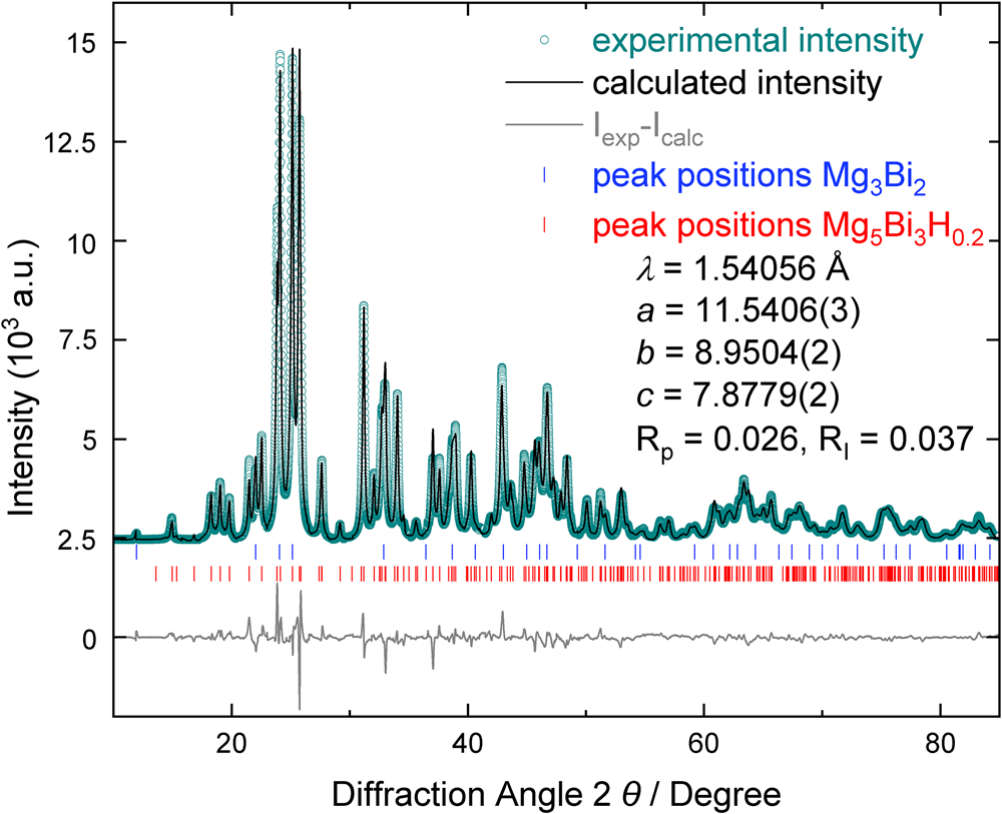
Powder X-ray diffraction diagram and refinement results based on full diffraction profiles of Mg_5_Bi_3_H_0.2_. Background of the measured diffraction pattern has been subtracted. The gray line on the bottom shows the difference between the calculated and the measured diffraction data. All intensities are shown on the same scale.

A two-phase refinement using full diffraction profiles confirms the presence of Mg_3_Bi_2_ and substantiates for Mg_5_Bi_3_H_x_ a structure model in the centrosymmetric space group *Pnma* (Fig. 3 and Supplementary Fig. 7). Admittedly, the position of hydrogen remains undetermined because of its weak scattering contribution in comparison to that of bismuth. Refinements with and without H atoms yield essentially the same results and *R*-values (compare Fig. 3 and Supplementary Fig. 6). Thus, the position of hydrogen is determined by quantum chemical optimization of the crystal structure (see subsection *Determination of the Hydrogen Position*) and included. Refinements of the Ca_5_Sb_3_F-type model^19^ converge to *R*_P_ = 0.026 and *R*_I_ = 0.037 (Tables 1 and 2; selected interatomic distances Mg−Bi and Mg−H are listed in Table S1). The resulting composition with respect to the metal atoms is in full accordance with the findings of the wavelength dispersive X-ray spectroscopy measurements. At the same *p*,*T* conditions, attempts to synthesize the new phase with a substantially higher hydrogen content (Mg_5_Bi_3_H_1_) by reaction of MgH_2_ + 9 Mg + 6 Bi yield a different product (see Supplementary Figs. 8 and 9).

**Table 1:** Experimental details of the powder X-ray diffraction measurement of Mg_5_Bi_3_H_0.2_ at room temperature as well as results of the least-squares refinements

Chemical composition	Mg_5_Bi_3_H_0.2_
Crystal system	Orthorhombic
Space group	*Pnma* (no.62)
Lattice parameters *a* / Å *b* / Å *c* / Å *V* / Å^3^	11.5406(3) 8.9504(2) 7.8779(2) 813.73(3)
Formula units per unit cell, *Z*	4
Calculated density *ρ*_calc_	6.11 g cm^−3^
Absorption coefficient, *µ*	126.63 cm^−1^
Measurement device	Huber Image Plate Guinier Camera G670 with transmission alignment
Radiation, *λ*	Cu*K*_α1_ radiation, 1.54056 Å
Function for profile fitting	Pseudo-Voigt
Measurement limits	4.0° ≤ 2*θ* ≤ 120° Refinement limits: 10.0° ≤ 2*θ* ≤ 85.4°
Number of reflections	365
Number of refined parameters	26
Structure refinement	Full profile with program package WinCSD
Residuals; GoF	*R*_p_ = 0.026, *R*_wp_ = 0.030, *R*_I_ = 0.037; 1.00

For further details, see deposited information CCDC 2334026.

**Table 2:** Atomic coordinates and isotropic displacement parameters (in Å^2^) of Mg_5_Bi_3_H_0.2_

Atom	Wyckoff Site		*x*	*y*	*z*	*B*_iso_ Å^2^
Mg1a	4*c*	0.765(1)		0.25	0.329(2)	0.94^a^
Mg2a	4*c*	0.2355(9)		0.25	0.636(2)	0.94^a^
Mg3a	4*c*	0.512(1)		0.25	0.481(1)	0.94^a^
Mg4a	8*d*	0.9205(7)		0.0595(7)	0.721(1)	0.94^a^
Bi1	4*c*	0.0208(1)		0.25	0.4262(2)	0.84(2)
Bi2	8*d*	0.67182(8)		0.02659(9)	0.5849(2)	0.94(1)
H^a, b, c^	4*c*	0.9012b		0.25b	0.8117b	1.5^a^

^a^Displacement parameter constrained for refinenement.

^b^Position form quantum chemical calculations and not refined.

^c^Site occupation factor 0.2 determined from chemical analysis.

### Chemical bonding analysis

In order to investigate the role of hydrogen in the high-pressure phase, we performed chemical bonding analysis for the idealized hydrogen-free case with composition Mg_5_Bi_3_ as well as for the scenario with interstitial hydrogen according to a hypothetical composition Mg_5_Bi_3_H_x_ (*x* = 1). The formal charge of a compound Mg_5_Bi_3_ may be rationalized as [Mg^+2^]_5_[Bi^−3^]_3_ × 1e^−^ pointing at an electron-excess compound, in which the cations offer more electrons than required in the anionic part^23^. Such a formal surplus of electrons is compatible with several different electronic scenarios. The excess electrons may fill antibonding states above the pseudo gap in the electronic density of states (DOS). Materials with these DOS features are interpreted as metallic Zintl phases^24–27^.

Another conceptual situation appears when the cationic component forms homoatomic bonds, e.g., in gallium mono-selenide comprising the gallium di-cation [Ga_2_]^4+^[(0b)Se^2−^]_2_. Alternatively, excess electrons may be localized in a lone pair of the cation, like in indium monobromide: [(lp)In^+^] [(0b)Br^−^]. And finally, a recent study on LuGe evidences that excess electrons Lu^3+^[(2b)Ge^2−^] × 1e^−^ are used for multi-atomic bonding and the formation of vertices-condensed polycations Lu_4_^28^.

**Fig. 4:**
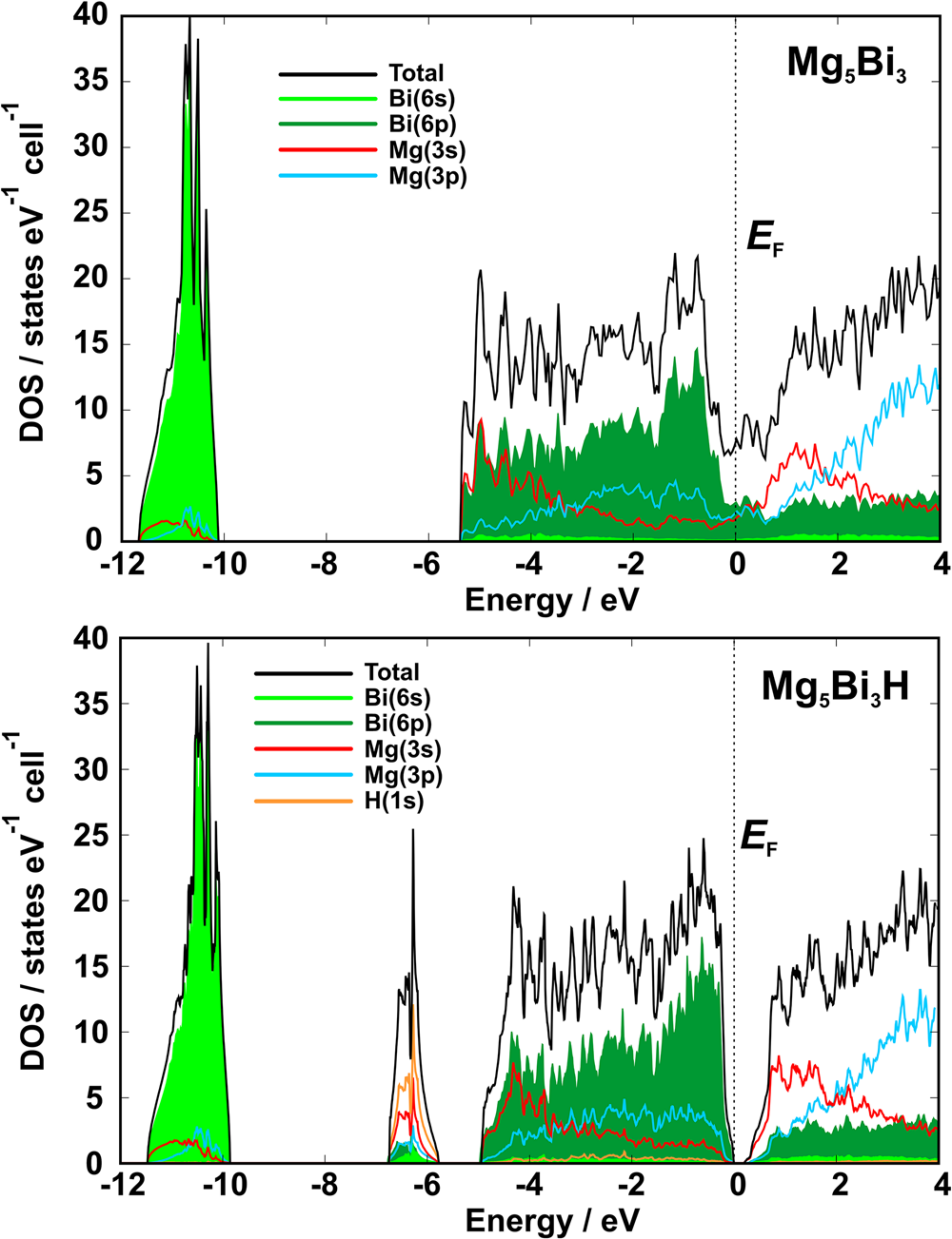
Calculated electronic density of states (DOS). (top) Hydrogen-free Mg_5_Bi_3_ and (bottom) idealized electron precise Mg_5_Bi_3_H. The total DOS is shown together with the essential contributions of partial states of Mg, Bi, and H.

To understand the situation of such an electron-excess model of the Mg_5_Bi_3_-type, the electronic density of states (DOS) is analyzed (Fig. 4, top). Below the Fermi level, it consists of two clearly separated regions. The first one (− 12 eV < *E* < − 10 eV) is mostly formed by *s* states of Bi with small contribution of Mg(*s*) and Mg(*p*). The main region (− 5.5 eV < *E* < *E*_F_) contains primarily the *p* states of bismuth mixed with *s* and *p* states of magnesium. A striking aspect is the position of the Fermi level slightly above the lowest section in the pseudo gap. Integration of the number of states between the pseudo-gap minimum and the Fermi level yields 3.87 electrons per unit cell, i.e., approximately one electron per formula unit, well in agreement with the electron count given above. Consequently, a compound Mg_5_Bi_3_ would bear a striking similarity to LuGe^28^.

That situation is fundamentally changed upon insertion of hydrogen. The magnesium *s* contributions located at the Fermi level in Mg_5_Bi_3_ are shifted to lower energies and form the separated DOS region together with mainly H(*s*) states in Mg_5_Bi_3_H (−7 eV < *E* < − 6 eV). As a result, a gap of approximately 0.2 eV appears at E_F_ (Fig. 4, bottom) in line with the electron precise balance [Mg^2+^]_5_[Bi^3−^]_3_[H^−^].

**Fig. 5:**
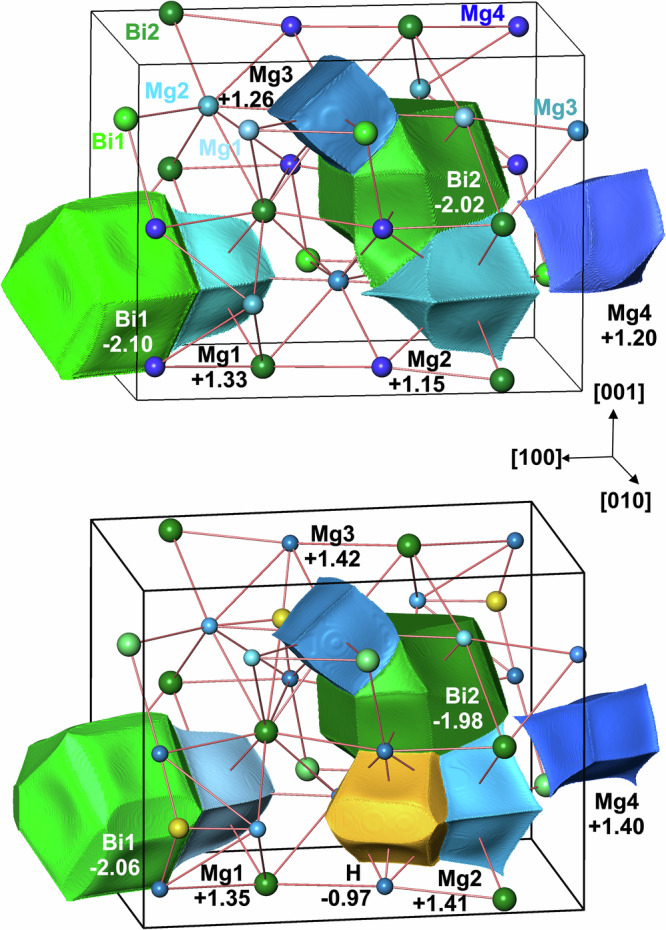
Calculated atomic shapes and effective charges according to the quantum theory of atoms in molecules (QTAIM). (top) Hydrogen-free Mg_5_Bi_3_ and (bottom) hypothetical electron-precise Mg_5_Bi_3_H. Here, the negative charge of −0.97 estimated for the hydrogen atoms clearly indicates the formation of a hydride anion.

Information about the bonding interactions are obtained from topological analysis of the calculated electron density (ED). The zero-flux surfaces in the gradient vector field of the ED form the boundaries of basins which represent atomic regions (Fig. 5, top).

While the bismuth charges of −2.02 and −2.10 for both crystallographic positions in Mg_5_Bi_3_ are quite similar, the spread of the magnesium atoms from +1.15 to +1.33 is slightly larger. The insertion of hydrogen in Mg_5_Bi_3_H (Fig. 5, bottom) mainly affects the calculated charge of only two magnesium atoms (Mg2 and Mg4) while the other values exhibit only minor changes. However, the changes effectively reduce the range to values between +1.35 and +1.42 in the hydride. The general increase is in line with the role of hydrogen as an additional electron acceptor with a calculated negative charge of −0.97.

The calculated charge transfers, in both Mg_5_Bi_3_ and Mg_5_Bi_3_H, are significantly lower than the formal oxidation states of +2 and −3 assigned to Mg and Bi, respectively. Nevertheless, the obtained effective charges for the Mg species comply with the general tendency of decreasing charge with increasing content of the cationic component. Similar trends are observed for Al in Al-Pt compounds^29^, for yttrium in Y-Ga^30^ and for Be in Be-Rh compounds^31^.

Further insight into the atomic interactions is obtained by analysis of the spatial distribution of the Electron-Localizability Indicator (ELI), in its ELI-D representation, combined with ED data within the electron-localizability approach. The bonds with Bi participation are mostly two- or three-atomic (with the exception of one four-atomic interaction Mg1-Mg3-Mg4-Bi2). Invariably, bismuth contributes the major part to the bond population, i.e., all those interactions are strongly polar. The bond basins around both bismuth atoms are mostly located within the atomic (QTAIM) shapes of the bismuth species (Supplementary Figs. 10 and 11 [top]), forming the anionic substructure in *hp*-Mg_5_Bi_3_. Summation of the populations of the basins around Bi1 and Bi2 yields 7.39 and 7.97 electrons, respectively. Ascribing these electrons to the Bi atoms results in effective charges *N*_val_^ELI^(Bi) of −2.61 for Bi1 and −2.97 for Bi2. The values are close to the conceptual one of −3. Usually, they are larger than estimated because of contributions of inner electrons to the valence bond basins^32^.

The ELI-D distribution in the hydride model Mg_5_Bi_3_H (Supplementary Fig. 12) is similar to that in Mg_5_Bi_3_. The ELI-D/QTAIM intersection method reveals pronounced polarity of the Mg-Bi and Mg-H bonds. Ascribing all electrons in the bond basins around Bi1, Bi2 and H (Supplementary Fig. 11, lower row) to the respective anion yields the *N*_val_^ELI^ values of 8.3, 8.2, and 2.08 electrons, respectively, corresponding to Bi^3−^ and H^−^. The finding is well in agreement with the conceptual values considering the effect mentioned above^32^.

**Fig. 6:**
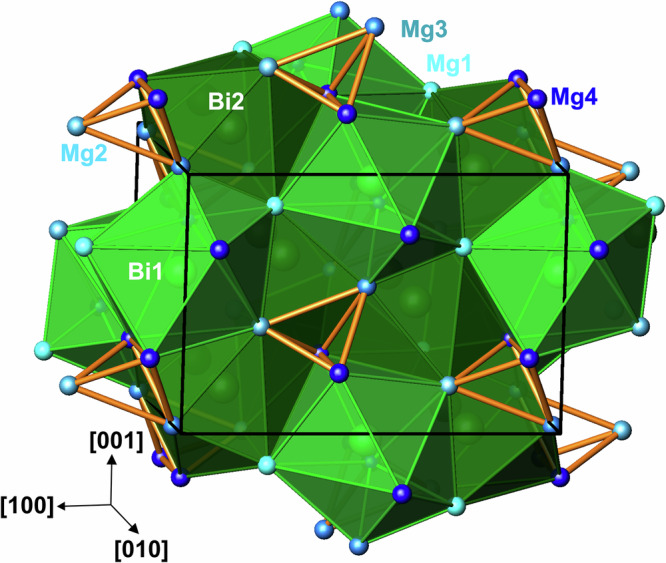
Crystal structure and bonding picture of Mg_5_Bi_3_ and Mg_5_Bi_3_H. Green polyhedrons [Mg_9_Bi] and [Mg_8_Bi] represent the anionic parts of the crystal structure. The fully transparent polyhedrons with orange edges indicate cationic [Mg_4_] clusters in the Mg_5_Bi_3_ model and [Mg_4_H] groups in the Mg_5_Bi_3_H archetype.

The most interesting bond basin is the one of the four-atomic interaction between the magnesium atoms Mg2-Mg3-Mg4-Mg4 (light pink in Supplementary Fig. 10, middle and bottom). It has one of the largest populations (1.42 e^−^) in the compound. The partial ELI-D technique^33^ shows that the electronic states between the pseudo-gap and the Fermi level contribute most to that feature. The isolated tetra-cations [Mg_4_] and the anionic species around Bi complete the bonding picture of Mg_5_Bi_3_ (Fig. 6).

### Determination of the hydrogen position

A known useful feature of the ELI (and the related electron localization function) is the ability to designate sites in crystal structures which are suitable locations for anions, specifically hydride^34–38^. Consequently, the ELI-D maximum pointing at the four-atomic bond between the magnesium atoms Mg2-Mg3-Mg4-Mg4 in Mg_5_Bi_3_ is identified as a potential position of the hydride ion in Mg_5_Bi_3_H. The negligible contribution of hydrogen to the X-ray scattering in bismuth compounds impedes the refinement of its coordinates by X-ray diffraction data. Consequently, the crystal structure including hydrogen is optimized using the experimental symmetry and lattice parameters. The finding that the resulting coordinates of the allocated hydride almost perfectly coincide with those of the fluorine position in Ca_5_Sb_3_F (a detailed comparison of atomic coordinates is shown in Table S2) impressively corroborates that the selected strategy represents an encouraging approach for intermetallic phases in general.

Consequently, the optimized atomic coordinates were used for the structure refinement with the X-ray diffraction data (see subsection *Synthesis and Characterization*) as well as for further quantum chemical calculations. Finally, the total energy for the reaction of Mg_5_Bi_3_ with elemental hydrogen to yield Mg_5_Bi_3_H_1_, was calculated according to the reaction equation

1 $${{{\rm{Mg}}}}_{5}{{{\rm{Bi}}}}_{3}+1/2{{\rm{H}}}_{2}\leftrightarrow {{{\rm{Mg}}}}_{5}{{{\rm{Bi}}}}_{3}{{\rm{H}}}.$$

Here, the hydride is found to be lower in energy by 48.8 kJ mol^−1^ (at 0 K), in excellent agreement with the experimentally observed phase formation.

## Methods

### Synthesis

Preparation and sample handling were performed in argon-filled glove boxes (MBraun, H_2_O < 0.1 ppm; O_2_ < 0.1 ppm). The precursor samples were prepared from elemental magnesium (Alfa Aesar, 325 mesh, 99.8%) and bismuth (Alfa Aesar, 200 mesh, 99.999%) powders in the molar ratio 5:3. The samples were well mixed in an agate mortar for about 5–10 min to assure homogeneity. This mixture is placed in boron nitride crucibles for extreme conditions synthesis.

High-pressure high-temperature experiments were realized using a hydraulic multi-anvil press^39^. Pressure transmission is realized in MgO octahedra with an edge length of 18 mm. Reaction temperatures are adjusted by resistance heating of graphite sleeves. Calibrations of pressure and temperature were done by observing resistance changes of bismuth^40^ and thermocouple-calibrated runs both having been conducted before the synthesis experiments. The compound Mg_5_Bi_3_H_X_ is synthesized at pressures between 4 and 5 GPa and within the temperature range of 973 to 1273 K. The maximal yield is obtained upon heating to 1073(50) K for 30 min followed by annealing at 773(30) K for three hours before quenching under load and decompression to ambient conditions.

### Powder X-ray diffraction

Sample characterization and data collection for structure refinement were realized in transmission alignment with a Huber image plate Guinier camera G670 with a beam size on the sample of 1 × 10 mm using Cu*K*α_1_ radiation, *λ* = 1.54056 Å All crystallographic calculations including lattice parameters and structure refinements on the basis of full diffraction profiles (Rietveld method) were performed using the WinCSD program package^41^.

### Metallography

Sample composition and phase distribution were analyzed by metallographic analysis. Light microscopy images (Zeiss Axioplan 2 with CCD Camera) at various magnifications were done followed by energy dispersive X-ray spectroscopy on a scanning electron microscope SEM (Jeol JSM 7800 F) with an attached energy-dispersive X-ray spectroscopy (EDXS) system (Quantax 400, Brucker, Silicon-Drift-Detector. Additionally, the wavelength dispersive X-ray spectroscopy analyses were performed with an electron microprobe (Cameca SX100 tungsten cathode). For these measurements, the selected sample surface was carefully prepared by argon-ion beam etching (Jeol IB 19520CCP). In order to avoid contamination of the clean surface by oxygen or water, the prepared specimen was transferred under inert conditions to the electron microprobe.

### Thermal analysis

Differential scanning calorimetry (DSC) experiments were done in a Netzsch DSC 404 C device using Al_2_O_3_ crucibles (up to 773 K) or in sealed Ta ampoules (up to 1123 K). Measurements were done with a heating and cooling rate of 10 K/min in argon atmosphere.

The hydrogen content in the various samples (educts and products) Mg, Bi, Mg_3_Bi_2_ and Mg_5_Bi_3_H_x_ was determined by releasing the hydrogen into the gas phase by means of thermal desorption, i.e., decomposition and subsequent qualitative and quantitative analysis of the gas phase.

The composition of the gas phase was analyzed by a temperature-dependent process using a TG-MS system, which is a combination of a thermo-balance STA 409 CD (NETZSCH) and a quadrupole mass-spectrometer QMS 422 (Pfeiffer Vacuum). The installation of this system in an argon-filled glove box (MBraun) enables handling and measurement of especially air- or moisture-sensitive samples.

The individual samples were measured using heating and cooling rates of 5 K/min and corundum crucibles in a Knudsen cell with Ta inlay, perforated lid (Effusion bore diameter 0.2 mm) and a thermocouple type S (PtRh/Pt). The measurements were carried out in flowing argon atmosphere as purging gas (Ar 99.999% 50 ml/min with subsequent drying and oxygen post-purification via a Big Oxygen Trap (Trigon Technologies). The detection of gas particles was carried out in MID mode for ions with m/z 1 (H^+^), 2 (H_2_^+^), 16 (O^+^), 17 (HO^+^), 18 (H_2_O^+^), 24 (Mg^+^), 32 (O_2_^+^) 209 (Bi^+^) with 70 eV electron impact ionization.

Educts and products were investigated in the temperature range from 298 to 813 K using sample masses of 28.48 and 12.45 mg (Mg), 17.13 mg (Bi), 35.51 mg (Mg_3_Bi_2_), as well as 23.91 and 30.25 mg of Mg_5_Bi_3_H_x_. Removal of hydrogen from elemental magnesium and bismuth was achieved under the same conditions and monitoring the hydrogen release.

The gases being released from the specimen were intermixed with a continuous argon purging-gas stream and directed to the ionization chamber of the mass spectrometer by using a skimmer located directly above the bore of the effusion crucible. Background mass spectra were recorded before the measurement by using the same conditions. We abstained from correcting the thermogravimetry data for buoyancy as this allows for a more suitable material combination of the crucible setup. The quantification of the released hydrogen was carried out by calibration using five independent measurements of NaH (Supplementary Fig. 3). The thermal decomposition of NaH shows only one sharp, well reproducible signal for the release of hydrogen into the gas phase between 250 and 400 °C with a maximum at 325 °C. Peak shape and baseline are ideally suited for using the hydrogen release during the thermal decomposition of NaH for quantitative calibration.

### Quantum chemical calculations

Electronic structure calculations and chemical bonding analysis were performed with the all-electron, local orbital full‒potential technique (FPLO) within the local density approximation^42^ (Perdew‒Wang parametrization^43^, scalar relativistic calculation, standard basis set, 12 × 12 × 12 k points). The experimentally obtained atomic coordinates were optimized keeping the experimental lattice parameters constant. For the analysis of chemical bonding in position space, the ED and the ELI-D were calculated with a specialized module implemented in the FPLO program package^44^. The topology of ED and ELI-D was analyzed with the program DGrid^45^. The ED was integrated within atomic basins, i.e., spatial regions confined by zero-flux surfaces in the gradient field of ED and ELI-D, respectively (for additional information, see Supplementary Methods). This technique represents the procedure proposed in the Quantum Theory of Atoms in Molecules (QTAIM^46^ and provides effective electron populations for the QTAIM atoms. Correspondingly, bond basins are the same type of confined regions associated to attractors in the valence region of the ELI-D gradient field. Further bonding relevant information is obtained from combined analysis of ED and ELI-D within the electron localizability approach^47^. 

## Supplementary information

Supplementary Material

Description of Additional Supplementary Files

Supplementary data

## Data Availability

The data sets generated during and analyzed during the current study are available from the corresponding author on reasonable request.

## Code availability

Differential calorimetry measurements are realized with NETZSCH Messung, version 6.1.0. and data are analysed using NETZSCH PROTEUS version 8.0.3. Thermogravimetry and mass spectroscopy measurement were done with the NETSCH Proteus software, version 6.1.0. (NETZSCH.com), and the Inficon AG Quadstar-32-bit software, version 7.02 (quadstar-32-bit.software.informer.com). For integration of the experimental charge, least squares fit of the calibration data and display of the experimental data as well as refinement results, we used OriginPro 2020b (64-bit, OriginLab Corporation). X-ray diffraction data are collected with G670 Imaging Plate Guinier Camera, version 6.0 build 86 by Huber Diffraktionstechnik GmbH (xhuber.com). WinCSD program package may be downloaded upon registration (wincsd.eu). The crystal structure is visualized with Atoms version 6.3.4. The FPLO package may be downloaded from www.fplo.de after registration. The ELI-D module for FPLO is available upon request from the authors. Please notice that German embargo regulations are in place for certain countries and institutions. Information on the AVIZO software for visualization of ELI-D and charge density is available at www.thermofisher.com.

## References

[1] Zhuravlev, N. N. & Melik Adamyan, V. R. Study of the crystal structure of the superconducting compounds SrBi_3_ and BaBi_3_. *Sov. Phys. Crystallogr.* **6**, 121–124 (1961).

[2] Iyo, A. et al. Large enhancement of superconducting transition temperature of SrBi_3_ induced by Na substitution for. *Sr. Sci. Rep.* **5**, 10089 (2015). 25965162 10.1038/srep10089 PMC4428034

[3] Winiarski, M. J. et al. Superconductivity in CaBi_2_. *Phys. Chem. Chem. Phys.* **18**, 21737–21745 (2016). 27435423 10.1039/c6cp02856j

[4] Zhang, H. et al. Topological insulators in Bi_2_Se_3_, Bi_2_Te_3_ and Sb_2_Te_3_ with a single Dirac cone on the surface. *Nat. Phys.* **5**, 438–442 (2009).

[5] Ren, Z., Taskin, A. A., Sasaki, S., Segawa, K. & Ando, Y. Large bulk resistivity and surface quantum oscillations in the topological insulator Bi_2_Te_2_Se. *Phys. Rev. B* **82**, 241306 (2010).

[6] Liu, Z. K. et al. Discovery of a three-dimensional topological Dirac semimetal, Na_3_Bi. *Science* **343**, 864–867 (2014). 24436183 10.1126/science.1245085

[7] Zintl, E. & Kaiser, H. Über die Fähigkeit der Elemente zur Bildung negativer Ionen. *Z. Anorg. Allg. Chem.* **211**, 113–131 (1933).

[8] Zintl, E. & Husemann, E. Bindungsart und Gitterbau binärer Magnesiumverbindungen. *Z. Physikal. Ch.* **B 21**, 138–155 (1933).

[9] Schäfer, H., Eisenmann, B. & Müller, W. Zintl phases: transitions between metallic and ionic bonding. *Angew. Chem. Int. Ed.* **12**, 693–712 (1973).

[10] Barnes, A. C., Guo, C. & Howells, W. S. Fast-ion conduction and the structure of *β*-Mg_3_Bi_2_. *J. Phys.: Condens. Matter* **6**, L467–L471 (1994).

[11] Schwarz, U. et al. CoBi_3_: a binary cobalt–bismuth compound and superconductor. *Angew. Chem. Int. Ed.* **52**, 9853–9857 (2013). 10.1002/anie.201302397 23881790

[12] Walsh, J. P. S. et al. MnBi_2_: a metastable high-pressure phase in the Mn−Bi system. *Chem. Mater.* **31**, 3083–3088 (2019).

[13] Walsh, J. P. S., Clarke, S. M., Meng, Y., Jacobsen, S. D. & Freedman, D. E. Discovery of FeBi_2_. *ACS Cent. Sci.* **2**, 867–871 (2016). 27924316 10.1021/acscentsci.6b00287 PMC5126710

[14] Clarke, S. M. et al. Discovery of a superconducting Cu–Bi intermetallic compound by high-pressure synthesis. *Angew. Chem. Int. Ed.* **55**, 13446–13449 (2016). 10.1002/anie.201605902 27666749

[15] Guo, K. et al. Weak interactions under pressure: hp-CuBi and its analogues. *Angew. Chem. Int. Ed.* **56**, 5620–5624 (2017). 10.1002/anie.201700712 28370908

[16] Powderly, K. M. et al. High-pressure discovery of *β*-NiBi. *Chem. Commun.* **53**, 11241–11244 (2017). 10.1039/c7cc06471c 28959808

[17] Altman, A. B. et al. Computationally directed discovery of MoBi_2_. *J. Am. Chem. Soc.* **143**, 214–222 (2021). 33372790 10.1021/jacs.0c09419

[18] Corbett, J. D. & Leon-Escamilla, E.-A. Role of hydrogen in stabilizing new hydride phases or altering old ones. *J. Alloy. Compd.* **356–357**, 59–64 (2003).

[19] Hurng, W.-M. & Corbett, J. D. Alkaline-earth-metal antimonides and bismuthides with the *A*_5_*Pn*_3_ stoichiometry. Interstitial and other Zintl phases formed on their reactions with halogen or sulfur. *Chem. Mater.* **1**, 311–319 (1989).

[20] Grube, G., Mohr, L. & Bornhak, R. Elektrische Leitfähigkeit und Zustandsdiagramm bei binären Legierungen. Das System Magnesium-Wismut. *Z. Elektrochem.* **40**, 143–150 (1934).

[21] Wobst, M. Das ternäre system Magnesium-Bismut-Zinn. *Z. Phys. Chem.* **219**, 239–265 (1962).

[22] Oh, C.-S., Kang, S.-Y. & Lee, D. N. Assessment of the Mg-Bi system. *Calphad* **16**, 181–191 (1992).

[23] Parthé, E. *Elements of Inorganic Structural Chemistry* Vol. 38 (Petit-Lancy, Switzerland, 1996).

[24] Parthé, E. Valence-electron concentration rules and diagrams for diamagnetic, non-metallic iono-covalent compounds with tetrahedrally coordinated anions. *Acta Crystallogr. B* **29**, 2808–2815 (1973).

[25] Zhao, J.-T. & Corbett, J. Square pyramidal clusters in La_3_ln_5_ and Y_3_In_5_. La_3_In_5_ as a metallic Zintl phase. *Inorg. Chem.* **34**, 378–383 (1995).

[26] Nesper, R. Structure and chemical bonding in Zintl-phases containing lithium. *Prog. Solid St. Chem.* **20**, 1–45 (1990).

[27] Nesper, R. Bonding patterns in intermetallic compounds. *Angew. Chem. Int. Ed.* **30**, 789–817 (1991).

[28] Freccero, R. et al. Excess” electrons in LuGe. *Angew. Chem. Int. Ed.* **60**, 6457–6461 (2021). 10.1002/anie.202014284 PMC7986909 33236821

[29] Baranov, A., Kohout, M., Wagner, F. R., Grin, Y. & Bronger, W. Spatial chemistry of the aluminum-platinum compounds: a quantum chemical approach. *Z. Kristallogr.* **222**, 527–531 (2007).

[30] Yu. Grin, A., Fedorchuk, R. J. & Wagner, F. R. Atomic charges and chemical bonding in Y-Ga compounds. *Crystals* **8**, 99 (2018).

[31] Agnarelli, L. et al. Charge transfer in Be-Ru compounds. *Chem. Eur. J*. **29**, e202302301 (2023). 10.1002/chem.202302301 37740670

[32] Freccero, R. et al. Polar-covalent bonding beyond the Zintl picture in intermetallic rare-earth germanides. *Chem. Eur. J.* **25**, 6600–6612 (2019). 30828887 10.1002/chem.201900510

[33] Wagner, F. R., Bezugly, V., Kohout, M. & Grin, Y. Charge decomposition analysis of the electron localizability indicator: a bridge between the orbital and direct space representation of the chemical bond. *Chem. Eur. J.* **13**, 5724–5741 (2007). 17458839 10.1002/chem.200700013

[34] Savin, A., Nesper, R., Wengert, S. & Fässler, T. F. ELF: the electron localization function. *Angew. Chem. Int. Ed.* **36**, 1808–1832 (1997).

[35] Lang, D. A. & Zaikina, J. V. Ca_2_LiC_3_H: a new complex carbide hydride phase grown in metal flux. *J. Am. Chem. Soc.* **132**, 17523–17530 (2010). 21090715 10.1021/ja107436n

[36] Al Alam, A. F. & Matar, S. F. Hydrogen insertion effects on the magnetic properties and chemical bonding within C14 Laves phases. *Prog. Solid State Chem.* **36**, 192–212 (2008).

[37] Matar, S. F. Intermetallic hydrides: a review with ab initio aspects. *Prog. Solid State Chem.* **38**, 1–37 (2010).

[38] Feng, X.-J. et al. Zintl-Phase Sr_3_LiAs_2_H: crystal structure and chemical bonding analysis by the electron localizability approach. *Chem. Eur. J.* **21**, 14471–1477 (2015). 26291332 10.1002/chem.201501236

[39] Walker, D., Carpenter, M. A. & Hitch, C. M. Some simplifications to multianvil devices for high pressure experiments. *Am. Mineral.* **75**, 1020–1028 (1990).

[40] Young, D. A. *Phase Diagrams of the Elements* (University of California Press, Berkeley, 1991).

[41] Akselrud, L. & Grin, Y. WinCSD: software package for crystallographic calculations (Version 4). *J. Appl. Crystallogr.* **47**, 803–805 (2014).

[42] Koepernik, K. & Eschrig, H. Full-potential nonorthogonal local-orbital minimum-basis band-structure scheme. *Phys. Rev. B* **59**, 1743–1757 (1999).

[43] Perdew, J. P. & Wang, Y. Accurate and simple analytic representation of the electron-gas correlation energy. *Phys. Rev. B* **45**, 13244–13249 (1992). 10.1103/physrevb.45.13244 10001404

[44] Ormeci, A., Rosner, H., Wagner, F. R., Kohout, M. & Grin, Y. Electron Localization Function in Full-Potential Representation for Crystalline Materials. *J. Phys. Chem. A* **110**, 1100–1105 (2006). 16420014 10.1021/jp054727r

[45] Kohout, M.* DGrid Versions 4.6−5.0* (Dresden, Germany, 2018−2021).

[46] Bader, R. F. W. *Atoms in Molecules-A Quantum Theory* 3rd edn. 222–237 (Oxford University Press, New York, 1990).

[47] Wagner, F. R. & Grin, Y. Chemical bonding analysis in position space. In *Comprehensive Inorganic Chemistry III* (eds. Reedijk, J. & Poeppelmeier, K. R.) 222–237 (Elsevier, 2023).

