# Publisher Correction: Locating hydrogen in the Mg_5_Bi_3_H_x_ Zintl phase

**DOI:** 10.1038/s42004-025-01616-w

**Published:** 2025-07-28

**Authors:** Teuta Neziraj, Lev Akselrud, Marcus Schmidt, Ulrich Burkhardt, Yuri Grin, Ulrich Schwarz

**Affiliations:** 1 Max-Planck-Institut für Chemische Physik fester Stoffe, Dresden, Germany; 2 Ivan Franko National University of Lviv, Lviv, Ukraine

**Keywords:** Solid-state chemistry, Chemical bonding

Correction to: *Communications Chemistry* 10.1038/s42004-025-01530-1, published online 30 April 2025

In the version of this article initially published, the subscript “2” was missing from Eq. (1), which has been corrected to read “Mg_5_Bi_3_ + 1/2H_2_ ↔ Mg_5_Bi_3_H” in the HTML and PDF versions of the article.

