# Supplementary material for: Locating hydrogen in the Mg5Bi3Hx Zintl phase
Source: Commun Chem. 2025 Apr 30;8:132. doi: 10.1038/s42004-025-01530-1 (PMC12043851; doi:10.1038/s42004-025-01530-1)
Supplement: Supplementary file 1 — Supplementary Material [file 42004_2025_1530_MOESM1_ESM.pdf]

Supplementary Data

Locating Hydrogen in the $Mg_5Bi_3H_x$ Zintl Phase

*Teuta Neziraj,[1] Lev Akselrud,[1,2] Marcus Schmidt,[1] Ulrich Burkhardt,[1] Yuri Grin,[1] Ulrich Schwarz[1]**

[1] Max-Planck-Institut für Chemische Physik fester Stoffe, Nöthnitzer Str. 40, 01187 Dresden, Germany

[2] Ivan Franko National University of Lviv, UA-79005 Lviv, Ukraine

*email: ulrich.schwarz@cpfs.mpg.de

Table of Contents

Supplementary Figure 1. Back scattering image of a metallographic sample of the product mixture Mg_3Bi_2 and $Mg_5Bi_3H_x$.

Supplementary Figure 2. Element mapping (Mg and Bi) of a phase mixture after heating the product mixture at ambient pressure.

Supplementary Figure 3. Calibration of ionic current of the mass spectrometer and amount of hydrogen.

Supplementary Figure 4. Evidence by mass spectroscopy for hydrogen in the magnesium which is used for synthesis.

Supplementary Figure 5. Powder X-ray diffraction diagram after thermal decomposition of the high-pressure product at ambient pressure.

Supplementary Figure 6. Powder X-ray diffraction diagram and refinement results based on full diffraction profiles for a medel with composition of Mg_5Bi_3.

Supplementary Figure 7. Powder X-ray diffraction diagram without background correction and refinement results based on full diffraction profiles of $Mg_5Bi_3H_{0.2}$.

Supplementary Figure 8. Powder X-ray diffraction diagram of the high-pressure phase prepared by reaction of 9 Mg + MgH_2 + 6 Bi.

Supplementary Figure 9. Hydrogen signal in the mass spectroscopy measurement of the high-pressure phase prepared by reaction of 9 Mg + MgH_2 + 6 Bi.

Supplementary Figure 10. ELI-D distribution and bond basins of Mg_5Bi_3.

Supplementary Figure 11. Bond basins of the bismuth atoms in Mg_5Bi_3 and in Mg_5Bi_3H.

Supplementary Figure 12. ELI-D distribution and bond basins of Mg_5Bi_3H.

Supplementary Table 1. Interatomic distances Mg-Bi and Mg-H in $Mg_5Bi_3H_x$.

Supplementary Table 2. Comparison of atomic positions in $Mg_5Bi_3H_x$ and Ca_5Sb_3F.

Supplementary Methods. Chemical bonding analysis.

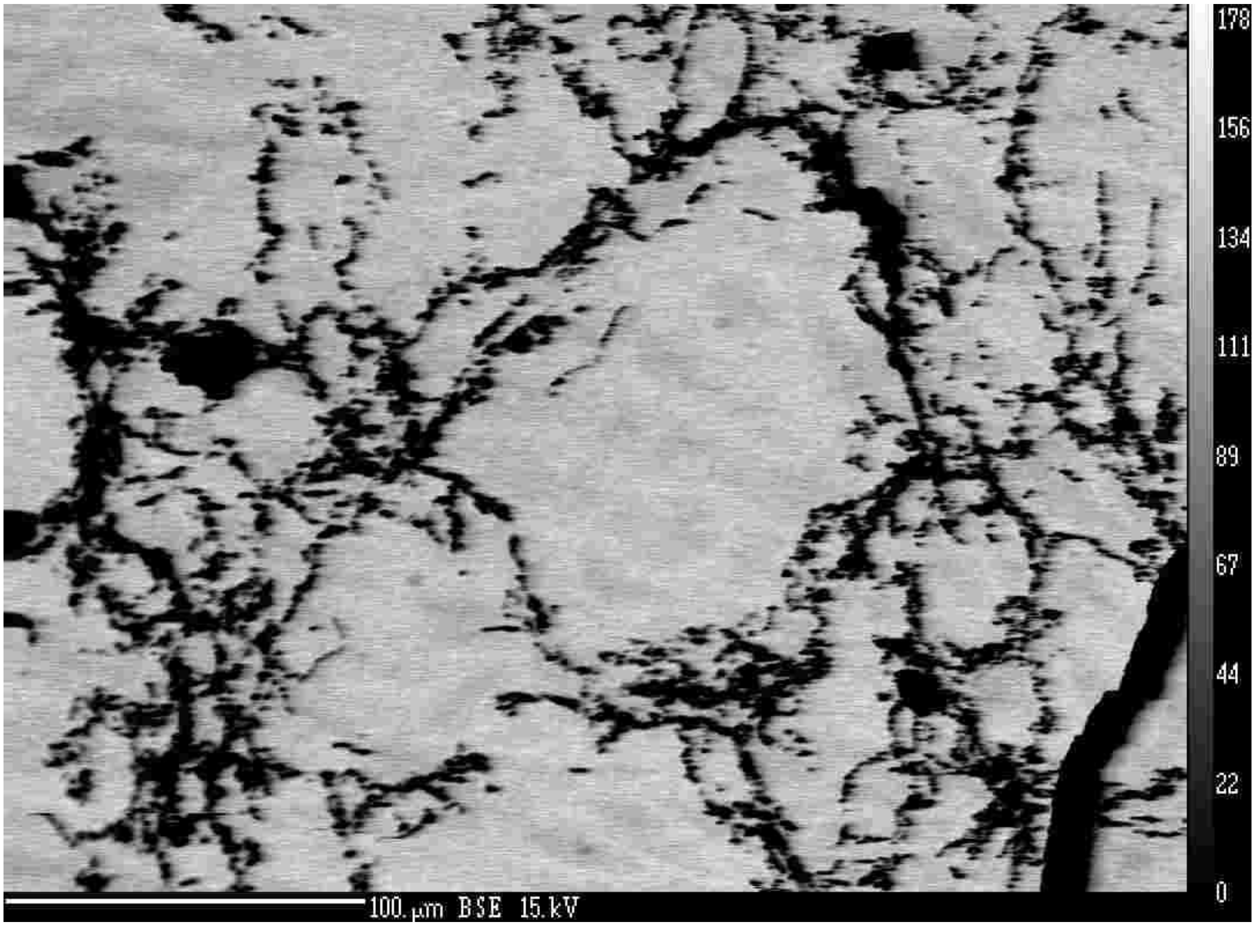

Supplementary Figure 1. Electron backscattering image of a metallographic sample of the reaction product. Black areas are cracks of the sample; the phases Mg_3Bi_2 and Mg_5Bi_3, respectively, adopt slightly different shades of grey. The homogeneity field of Mg_3Bi_2 covers a range of Mg:Bi ratios, which includes the composition of Mg_5Bi_3 at the reaction temperature.

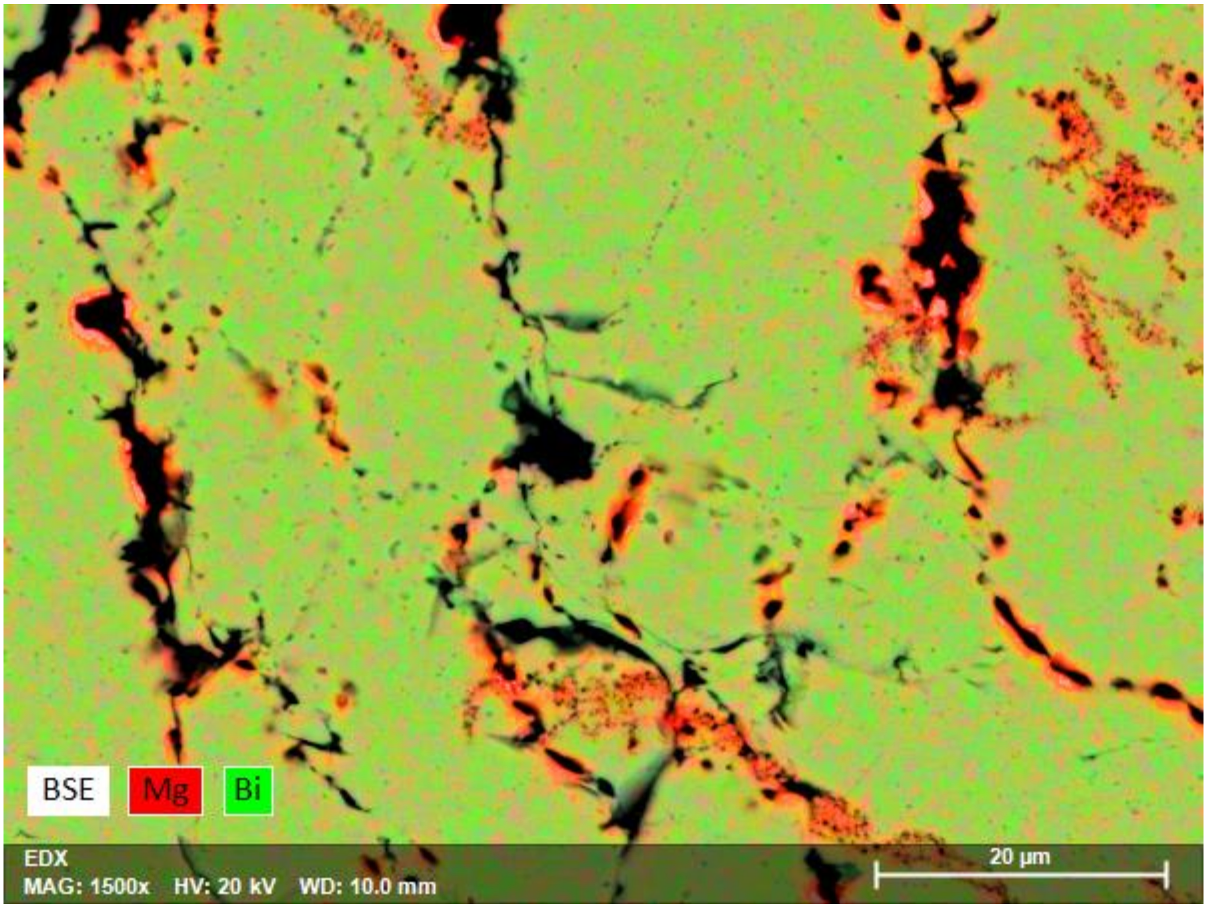

Supplementary Figure 2. Electron backscattering image of the product after heating to 770 K at atmospheric pressure, which induces transformation into Mg_3Bi_2 and magnesium being the stable phases at ambient conditions. Energy-dispersive X-ray spectroscopy is used for the superimposed element mappings of magnesium (red) and bismuth (green). Cracks of the sample show in black; the intense orange regions are attributed to segregated magnesium-rich phase mixtures.

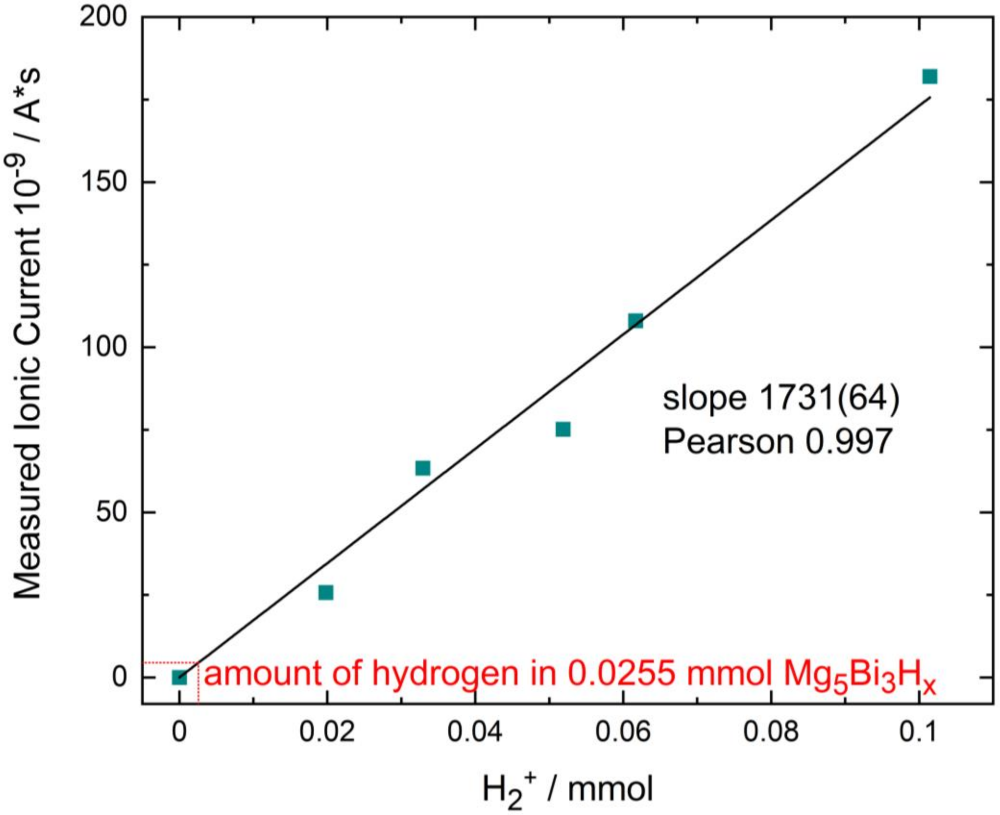

Supplementary Figure 3. Ionic current of the mass spectrometer as a function of the hydrogen amount released by thermal decomposition of NaH. The experimental data points (turquoise squares) are used for calibration; the result of the linear least squares fit (black line) is used for the determination of hydrogen in the synthesized sample of $Mg_5Bi_3H_{0.2}$ (dotted red line).

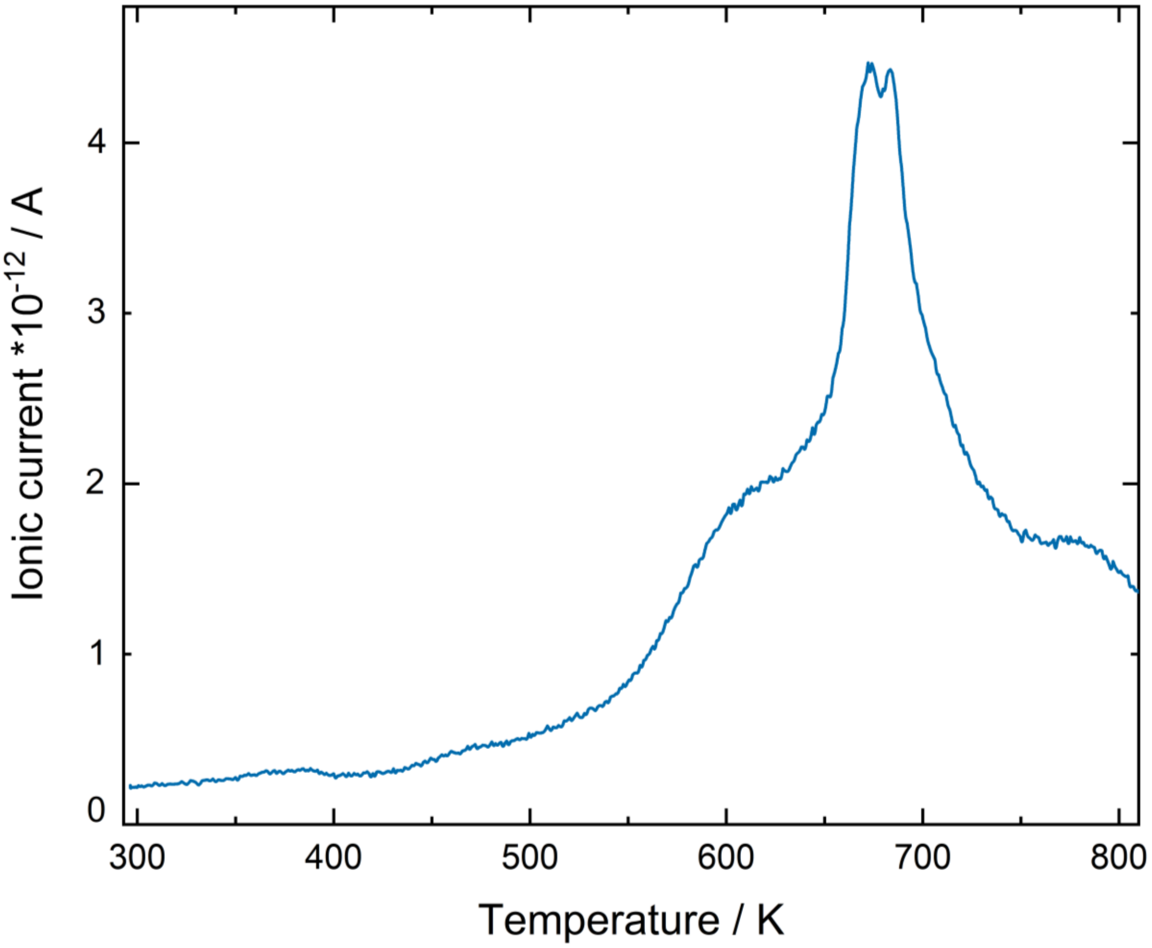

Supplementary Figure 4. Heating of the elemental magnesium used for synthesis and hydrogen signal H_2^+ of the mass spectrometer. The measured ionic current of the H_2^+ species as a function of temperature is shown in blue.

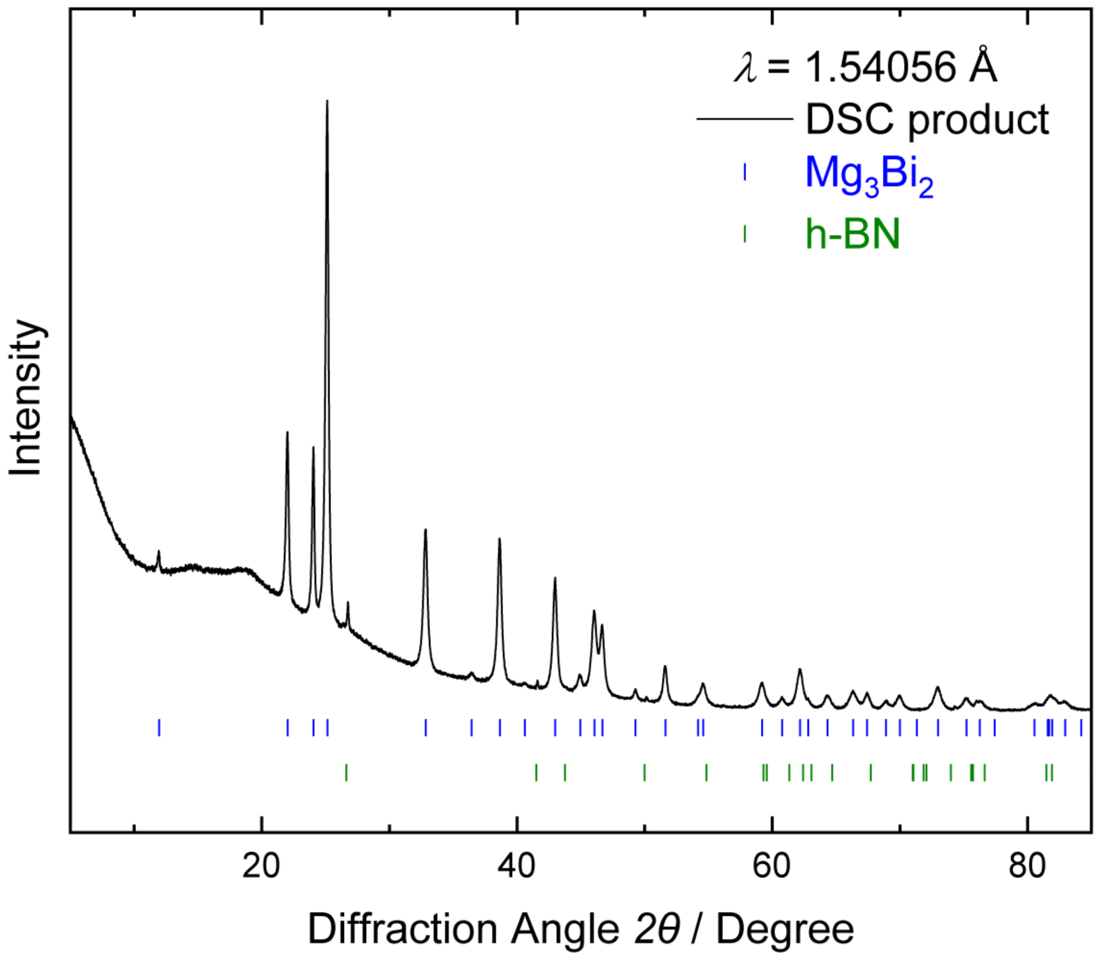

Supplementary Figure 5. Powder X-ray diffraction diagram of the product after heating to 770 K, i.e. well above the exothermal decomposition and below the melting of the eutectic mixture. The data point at the transformation into Mg_3Bi_2, a phase with considerable homogeneity range. Extra lines are assigned to small amounts of the crucible material hexagonal boron nitride. Measurement of the powder X-ray diffraction data is done with a Huber image plate Guinier camera G670 operated with CuKα1 radiation.

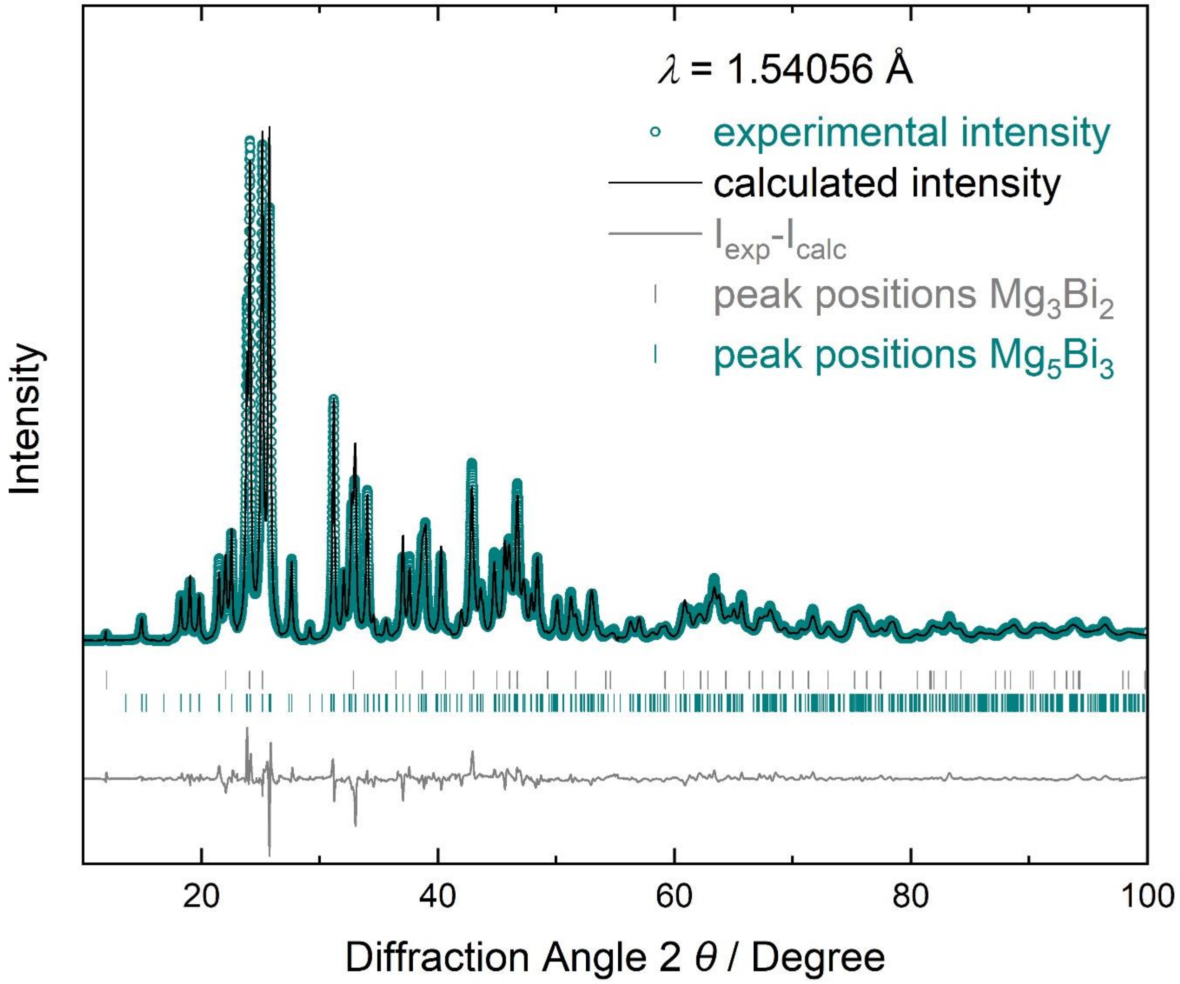

Supplementary Figure 6. Powder X-ray diffraction diagram of the high-pressure product plus refinement results using an Yb_5Sb_3-type structure model, i.e., including the positions of magnesium and bismuth only without suggesting a location for hydrogen because of its minute scattering contribution. Measurement of the powder X-ray diffraction data is done with a Huber image plate Guinier camera G670 operated with CuKα1 radiation. Background of the experimental data has been subtracted.

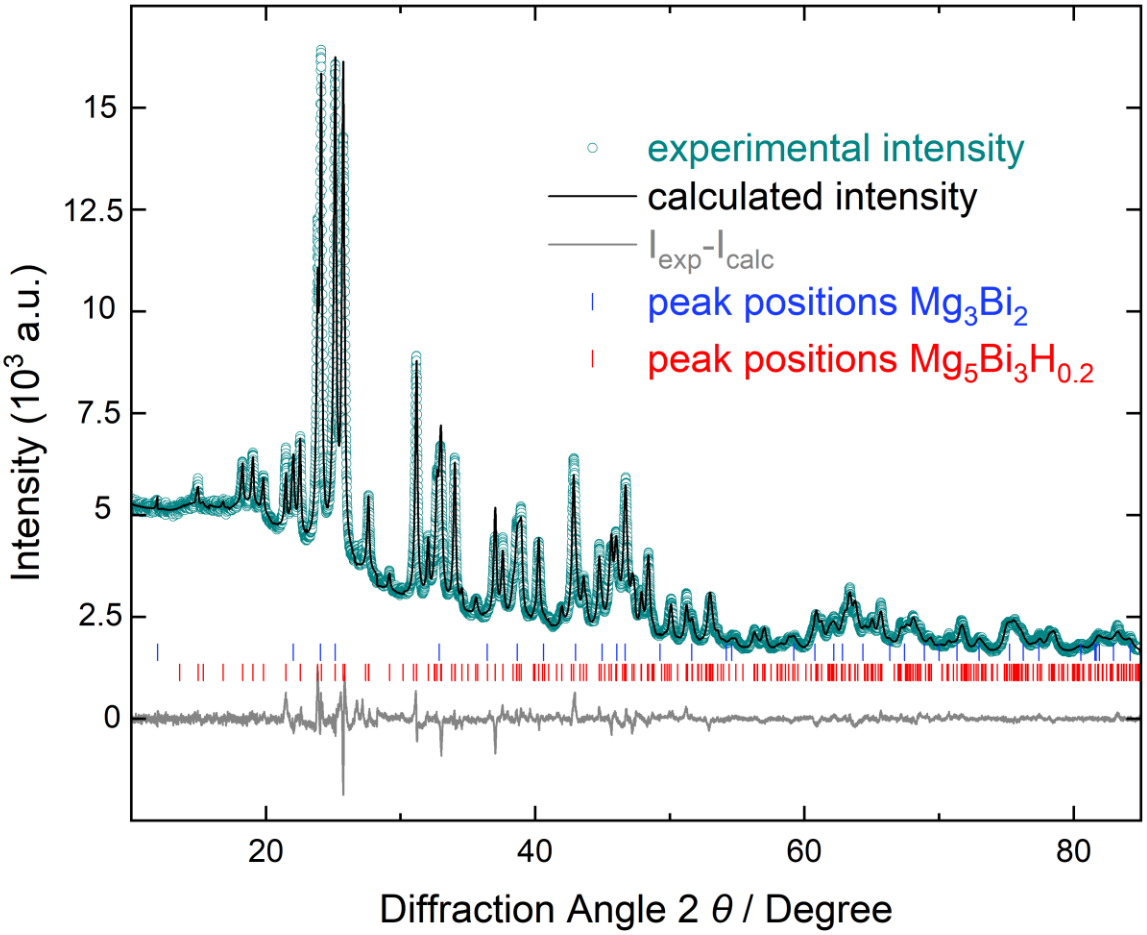

Supplementary Figure 7. Powder X-ray diffraction diagram and refinement results based on full diffraction profiles of $Mg_5Bi_3H_{0.2}$. All intensities are shown on the same scale. Measurement of the powder X-ray diffraction data is done with a Huber image plate Guinier camera G670 operated with CuKα1 radiation.

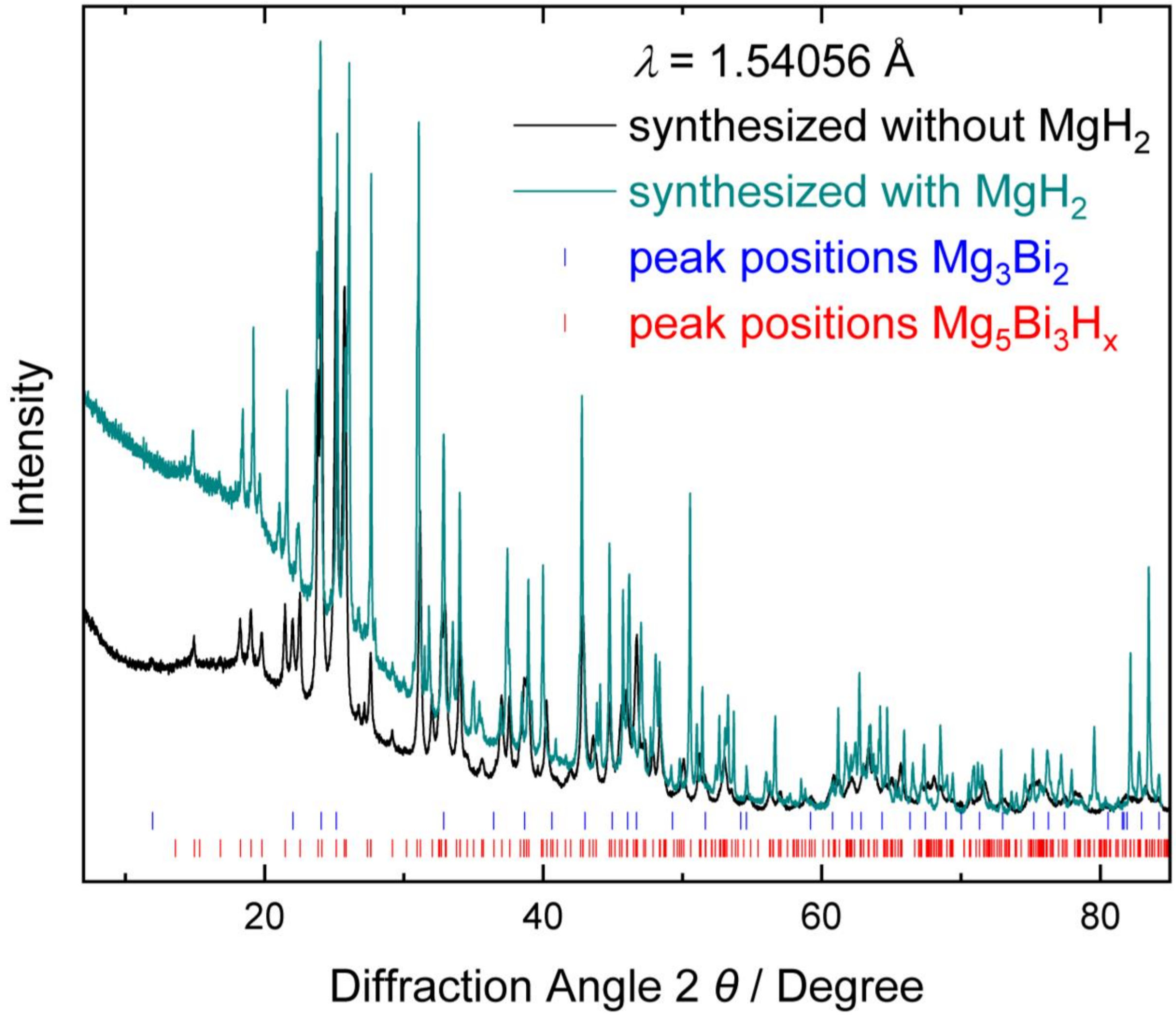

Supplementary Figure 8. Powder X-ray diffraction diagram of the high-pressure product prepared by reaction of MgH_2 with Mg and Bi aiming at the synthesis of $Mg_5Bi_3H_1$ in comparison to that of $Mg_5Bi_3H_{0.2}$. Additional reflections and markedly changed intensities indicate major differences of the phases. Measurement of the powder X-ray diffraction data is done with a Huber image plate Guinier camera G670 operated with CuKα1 radiation.

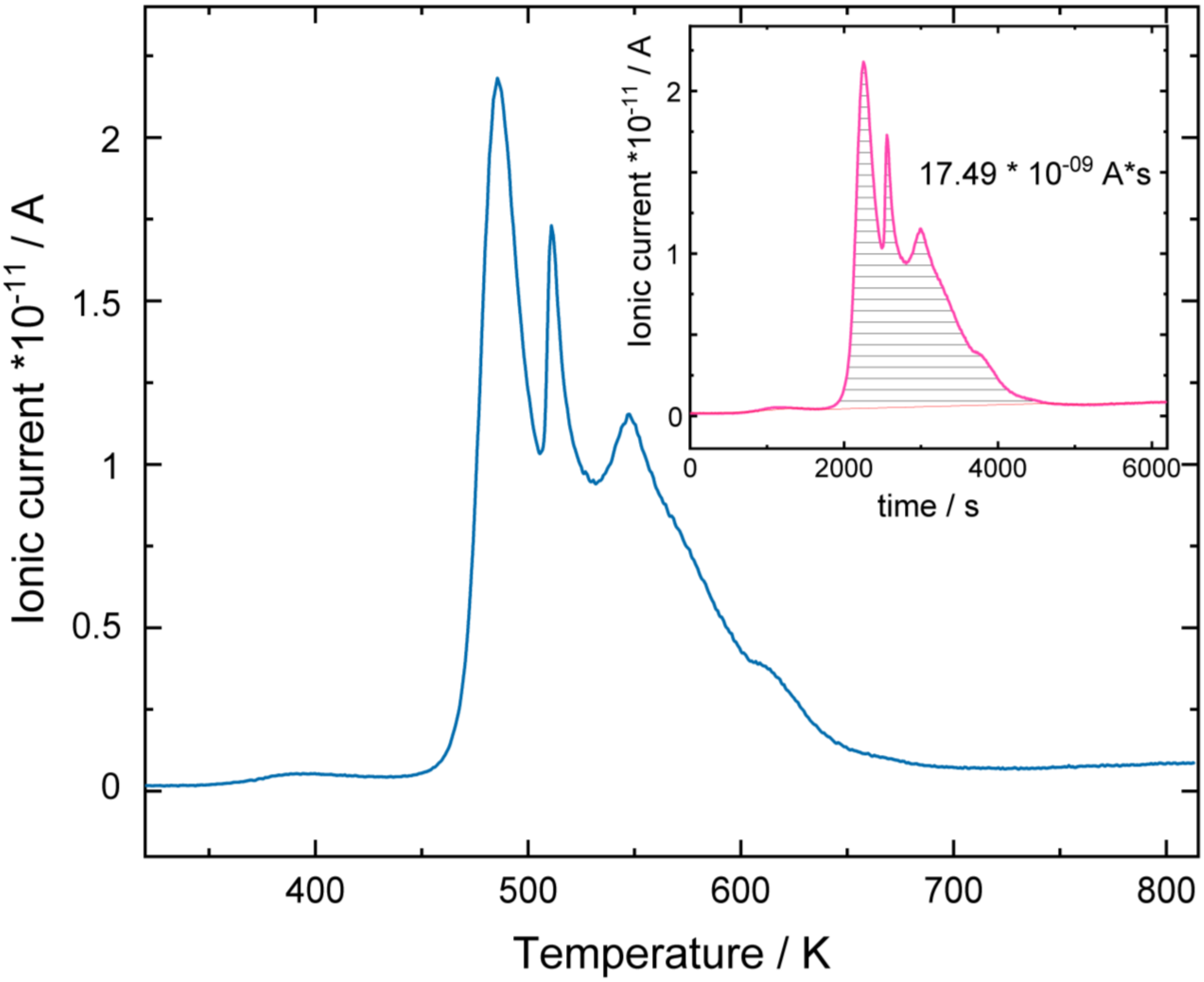

Supplementary Figure 9. Measurement of the product from a high-pressure synthesis using MgH_2, Mg and Bi. The signal indicates the ionic current by H_2^+ of the mass spectrometer. (Main panel) Measured ionic current as a function of temperature. (Inset) Signal of the ionic current as a function of time and integral (shaded area). The unknown product exhibits four signals at different temperatures in comparison to one signal for $Mg_5Bi_3H_{0.2}$ (see Figure 1 in main text) confirming significant structural differences of the phases.

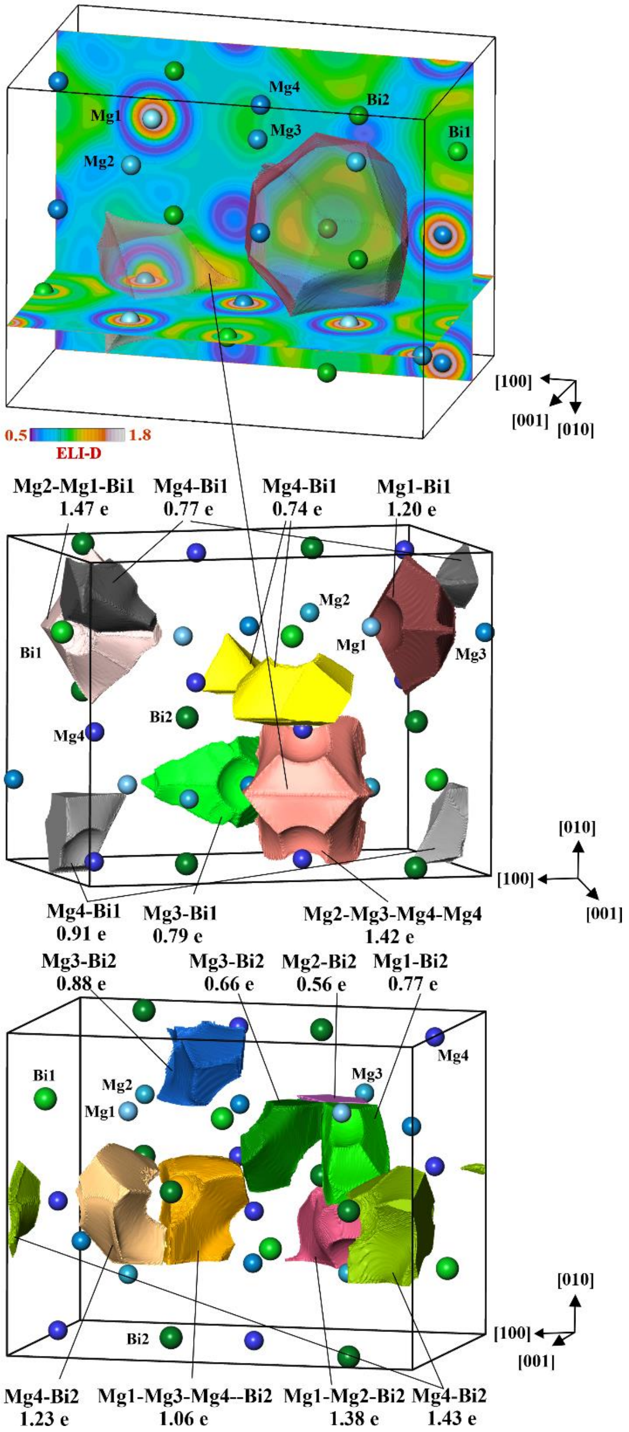

Supplementary Figure 10. Electron Localizability Indicator in *hp*-Mg_5Bi_3: (top) ELI-D distribution in the (*x* ¼ *z*) and (*x y* ≈⅓) planes with the atomic shapes (transparent) of Mg1 (left) and Bi2 (right). (middle and bottom) ELI-D bond basins and their populations.

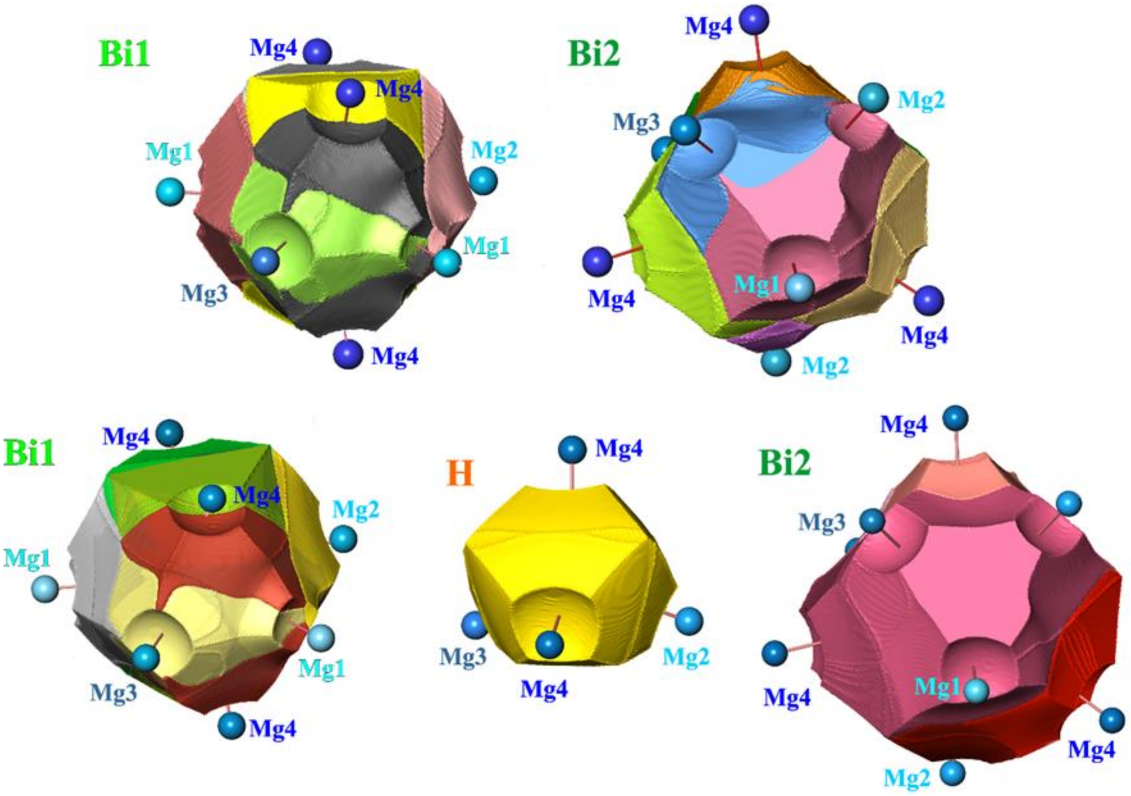

Supplementary Figure 11. Bond basins around the Bi1 and Bi2 atoms in Mg_5Bi_3 (top) and around Bi1, Bi2 and H atoms in Mg_5Bi_3H (bottom). Color codes for Mg_5Bi_3 for Mg_5Bi_3H are consistent with those used in the Supplementary Figures 10 and 12, respectively.

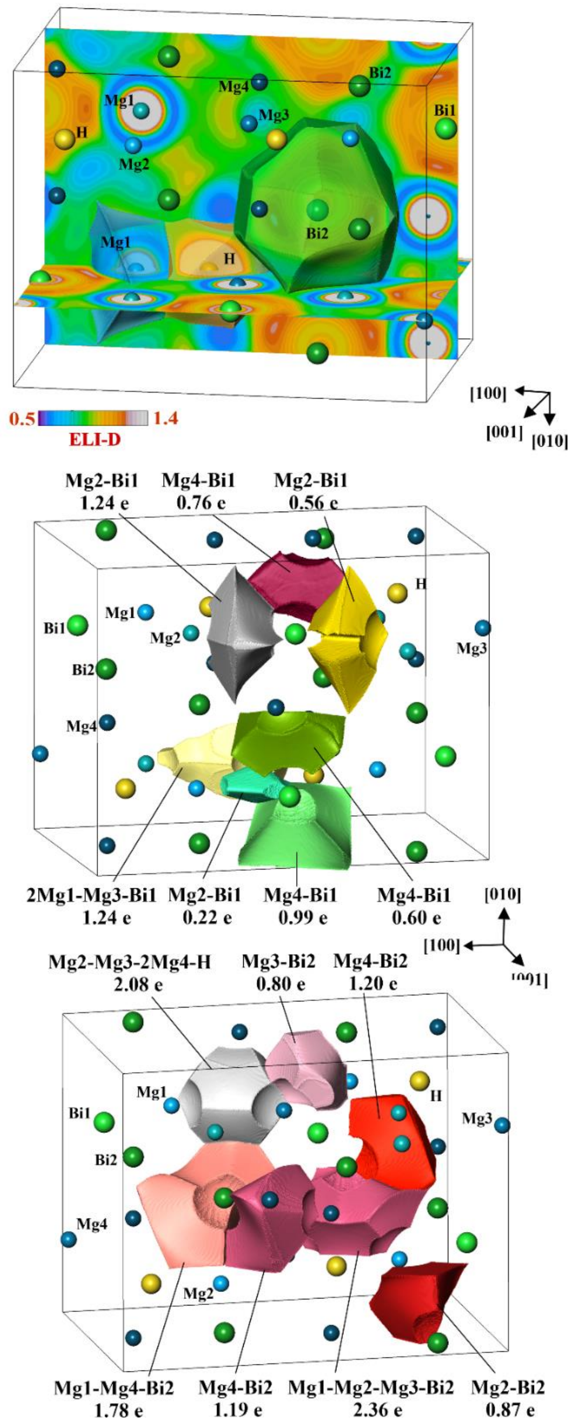

Supplementary Figure 12. Electron Localizability Indicator in *hp*-Mg_5Bi_3H: (top) ELI-D distribution in the (*x* ¼ *z*) and (*x y* ≈⅓) planes with the atomic shapes (transparent) of Mg1, H and Bi2. (middle and bottom) ELI-D bond basins and their populations.

Supplementary Table 1. Selected interatomic distances (in Å) for $Mg_5Bi_3H_x$.

Bi1	Mg1	3.05(1)
	Mg1	3.46(1)
	Mg2	2.98(1)
	Mg3	3.21(1)
	Mg4 (2x)	3.079(7)
	Mg4 (2x)	3.105(8)
Bi2	Mg1	3.036(8)
	Mg1	3.218(7)
	Mg2	3.062(8)
	Mg2	3.210(7)
	Mg3	2.841(8)
	Mg3	3.301(8)
	Mg4	3.078(8)
	Mg4	3.154(8)
	Mg4	3.292(8)
H	Mg2	1.956
	Mg3	2.074
	Mg4 (2x)	1.862

Supplementary Table 2. Atomic coordinates of $Mg_5Bi_3H_x$ and the Ca_5Sb_3F-type model [19] in the standardized setting. For the comparison, the origin of $Mg_5Bi_3H_x$ has been shifted by 1/2,1/2,0 before transforming the coordinates to the appropriate equivalent position. Only significant digits are given.

Wyckoff	Atom in Mg_5Bi_3H	x<br>y<br>z	Positional parameters	Atom in Ca_5Sb_3F	Positional parameters
4c	Mg1		0.235<br>¼<br>0.829	Ca2	0.229<br>¼<br>0.8229
4c	Mg2		0.2645<br>¼<br>0.364	Ca4	0.2855<br>¼<br>0.3561
4c	Mg3		0.998<br>¼<br>0.519	Ca3	0.0074<br>¼<br>0.5444
8d	Mg4		0.0795<br>0.0595<br>0.221	Ca1	0.07273<br>0.0422<br>0.1933
4c	Bi1		0.4792<br>¼<br>0.5738	Sb1	0.48159<br>¼<br>0.58229
8d	Bi2		0.32818<br>0.02659<br>0.0849	Sb2	0.32963<br>0.01878<br>0.07525
4c	H		0.0988<br>¼<br>0.3117	F	0.1036<br>¼<br>0.3029

Supplementary Methods

Quantum chemical bonding analysis

The zero-flux surfaces in the gradient vector field of the electron density in Mg_5Bi_3 form the boundaries of electron density basins which represent atomic regions within the framework of the Quantum Theory of Atoms in Molecules (QTAIM [S1]). Typical cations in the QTAIM representation show rather spherical shapes. More plane faces appear if the electronegativity difference between the bond partner is smaller, this indicates lower polarity of the interactions [S2]. The bismuth species in Mg_5Bi_3 (Figure 6) show large shapes with slightly concave faces toward the magnesium ligands, well in agreement with the calculated charge transfer. The shapes of the magnesium species deviate significantly form from spherical symmetry and show convex faces toward the neighboring bismuth ligands, while the faces toward magnesium neighbors for Mg2, Mg3 and Mg4 remain rather plane already indicating homoatomic bonding.

Integration of electron density in spatial regions, defined in QTAIM yields their electronic populations. The subtraction of those values from the according atomic numbers results in QTAIM effective atomic charges.

Due to chemical bonding, the spherical distribution of ELI-D, which is characteristic for non-interacting atoms, is violated and attractors (maxima) may appear in the regions of valence or penultimate shells, signaling bonding and indicating its geometrical location [S4]. In case of Mg_5Bi_3 and Mg_5Bi_3H, the penultimate shells of both, Mg and Bi, are still spherical (Supplementary Figures 4 and 6, top) which agrees with the dominant role of the s and p electrons in the electronic DOS. The ELI-D maxima appear in the valence shells (cf. position of maxima with respect to the Mg1 and Bi2 atomic shapes (Supplementary Figures 4 and 6, top). Each of the so-formed attractors (maxima) has its own ELI-D basin, which is determined, like the QTAIM atomic basins by the zero-flux surfaces in the gradient vector field of ELI-D. The number of common surfaces of a bonding basin with the attached core basins of the penultimate shell defines the atomicity of the bonding basin and characterizes the number of atomic species participating in this bond (bond atomicity). The common surfaces with the attached core basins are pronouncedly concave (Supplementary Figures 4 and 6, middle and bottom).

References

[S1] R. F. W. Bader, *Atoms in Molecules-A Quantum Theory*, Oxford University Press, New York, 3rd Ed. 1990, 222-237.

[S2] F. R. Wagner, Yu. Grin, Chemical bonding analysis in position space. In: Comprehensive Inorganic Chemistry III (Third Edition) Elsevier, 2023, p. 222-237.
