# Supplementary material for: Locating hydrogen in the Mg5Bi3Hx Zintl phase
Source: Commun Chem. 2025 Apr 30;8:132. doi: 10.1038/s42004-025-01530-1 (PMC12043851; doi:10.1038/s42004-025-01530-1)
Supplement: Supplementary file 2 — Description of Additional Supplementary Files [file 42004_2025_1530_MOESM2_ESM.pdf]

Description of Additional Supplementary Files

File name: Supplementary Data
Description: cif file of crystal structure
